# Association between Osteoporosis and Meniere’s Disease: Two Longitudinal Follow-Up Cohort Studies

**DOI:** 10.3390/nu14224885

**Published:** 2022-11-18

**Authors:** Hyo Geun Choi, Juyong Chung, Dae Myoung Yoo, Chang Ho Lee, So Young Kim

**Affiliations:** 1 Department of Otorhinolaryngology-Head & Neck Surgery, Hallym University College of Medicine, Anyang 14068, Republic of Korea; 2 Department of Otorhinolaryngology-Head & Neck Surgery, Wonkwang University School of Medicine, Iksan 54538, Republic of Korea; 3 Hallym Data Science Laboratory, Hallym University College of Medicine, Anyang 14068, Republic of Korea; 4 Department of Otorhinolaryngology-Head & Neck Surgery, CHA Bundang Medical Center, CHA University, Seongnam 13496, Republic of Korea

**Keywords:** Meniere’s disease, osteoporosis, risk factors, cohort studies, epidemiology

## Abstract

A high rate of Meniere’s disease (MD) in patients with osteoporosis has been suggested. This research intended to estimate the bidirectional association of MD with osteoporosis. The ≥40-year-old population in the Korean National Health Insurance Service-Health Screening Cohort 2002–2019 was examined. In study I, 9529 patients with MD and 38,116 control I participants were analyzed for a previous history of osteoporosis. In study II, 65,858 patients with osteoporosis and 65,858 control II participants were analyzed for a previous history of MD. Stratified Cox proportional hazard models were applied to calculate the hazard ratios (HRs) and 95% confidence intervals (CIs) of MD for osteoporosis in study I and of osteoporosis for MD in study II. The rate of a prior history of osteoporosis was 13.3% for the MD group and 11.3% for the control I group. The patients with MD had a 1.12 times higher HR for previous osteoporosis (95% CI = 1.04–1.20). In study II, the rate or a prior history of MD was 3.7% for patients with osteoporosis and 2.0% for the control II group. The patients with osteoporosis had a 1.50 times higher HR for previous MD (95% CI = 1.40–1.61). Most subgroups according to age, sex, and comorbid conditions demonstrated consistent bidirectional associations between MD and osteoporosis. Adult patients with MD had a greater risk of osteoporosis. In addition, adult patients with osteoporosis also showed a higher risk of MD.

## 1. Introduction

Meniere’s disease (MD) is an inner ear disease with relapsing cochleovestibular symptoms of vertigo, ear fullness, and hearing loss [1]. Approximately 50–200/100,000 adults were reported to suffer from MD [2]. The etiology of MD has been suggested to be multifactorial, including an autoimmune dysfunction, a viral infection, and genetic factors [3,4,5]. These etiologic causes have been presumed to induce hydrops of the endolymphatic duct, which has been acknowledged as a main pathophysiologic mechanism of MD [6]. In addition, a few studies have suggested that otoconia that detach from the macula of the saccule and obstruct the ductus reuniens or endolymphatic duct can contribute to the development of MD [7]. Thus, it can be postulated that the derangement or degeneration of otoconia may increase the risk of MD. Because osteoporosis is one of the factors related to the degeneration of otoconia, osteoporosis can be a risk factor for MD.

The homeostasis of calcium is associated with the regulation of otoconia in animal studies, since a high risk of benign paroxysmal positional vertigo (BPPV) in patients with osteoporosis has been reported [8,9]. In addition, osteoporosis was linked with an increased risk of vestibular dysfunction (adjusted odds ratio = 2.47, 95% confidence intervals [95% CI] = 1.05–5.81) [10]. It was suggested that the demineralization of the vestibular labyrinth and elevated free calcium in the endolymphatic flow can induce vestibular dysfunction in patients with osteoporosis [10]. However, the causality between vestibular dysfunction and osteoporosis has not been explored. In addition to the potential impact of osteoporosis on vestibular impairment, it has been presumed that vestibular dysfunction can disturb bone homeostasis [11]. Therefore, the risk of osteoporosis in patients with vestibular dysfunction can be postulated.

We hypothesized that osteoporosis can be related to a greater risk of MD and that patients with MD can have a greater risk of osteoporosis. Although the exact pathophysiologic mechanism has not been elucidated, osteoporosis has been indicated as a risk factor for vestibular dysfunction [10]. In addition, prior studies proposed the possible association of osteoporosis with cochlear dysfunction [12]. Because the dysfunction of the cochlea-vestibular system is related to MD, we supposed that osteoporosis can be associated with the risk of MD. Moreover, MD can induce osteoporotic changes via defects in the vestibular input to the brainstem and influence the sympathetic function [11]. To examine this supposition, two independent case–control studies were conducted and analyzed the risk of osteoporosis in patients with MD and vice versa. This study is novel to investigate the bidirectional association between MD and osteoporosis.

## 2. Methods

### 2.1. Ethical Considerations

The ethics committee of Hallym University (2019-10-023) permitted the analyses and exempted the authors from obtaining a written informed consent for the current research. This study analyzed the ≥40-year-old population in the Korean National Health Insurance Service-Health Screening Cohort 2002–2019. This study complied with the STROBE guidelines.

### 2.2. Diagnostic Criteria

Osteoporosis and MD were classified as in previous studies [13,14]. Osteoporosis was classified based on the international classification of diseases (ICD)-10 codes (M80, M81, and M82), 2 or more times of clinical visits, and the examination of bone density using X-ray or computed tomography [13].

MD was classified based on the ICD-10 codes (H810), 2 or more times of clinical visits, and the examination of pure tone audiometry [14].

### 2.3. Study I

**Figure 1:**
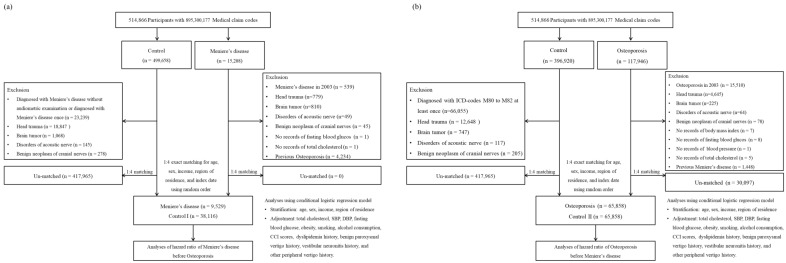
(**a**) A schematic illustration of the participant selection process that was used in the present study. Of a total of 514,866 participants, 9529 Meniere’s disease participants were matched with 38,116 control participants for age, sex, income, and region of residence. (**b**) A schematic illustration of the participant selection process that was used in the present study. Of a total of 514,866 participants, 65,858 osteoporosis participants were matched with 65,858 control participants for age, sex, income, and region of residence.

We enrolled 15,208 patients with MD. In total, 499,658 control participants were identified who were not diagnosed with MD during 2002–2019. Among the patients with MD, 539 patients who were diagnosed in 2002, as well as 779 MD patients and 18,847 control participants who had a history of head trauma were excluded. Participants with a history of brain tumors, disorders of acoustic nerves, and benign neoplasms of cranial nerves were also excluded. Age, sex, income, and region of residence were matched between the MD patients and the control participants. Finally, 9529 MD participants and 38,116 control I participants were included (Figure 1).

### 2.4. Study II

We identified 117,946 osteoporosis patients, as well as 396,920 control participants who were not diagnosed with osteoporosis during 2002–2019. Then, 15,510 osteoporosis patients who were diagnosed in 2002 and 4645 osteoporosis patients and 12,648 control participants who had a history of head trauma were excluded. Participants with a history of brain tumors, disorders of acoustic nerves, and benign neoplasms of cranial nerves were excluded too. Age, sex, income, and region of residence were matched between the osteoporosis patients and the control participants. Finally, 65,858 osteoporosis participants and 65,858 control II participants were included (Figure 1).

### 2.5. Variables

The variables of age, income level, region of residence, smoking, alcohol consumption, obesity, Charlson Comorbidity Index (CCI) were classified as previously described [15]. Ten age groups were defined, i.e., 40–44, 45–49, 50–54, 55–59, 60–64, 65–69, 70–74, 75–79, 80–84, and 85+ years of age. Five income groups were specified, from class 1 (lowest) to 5 (highest). Two groups of region of residence were defined as urban and rural areas. The status of smoking was defined as nonsmoker, past smoker, and current smoker based on a self-reported questionnaire. The frequency of alcohol consumption was defined as <1 time a week and ≥1 time a week. The body mass index (BMI, kg/m^2^) groups were classified as <18.5 (underweight), from.5 to <23 (normal), from 23 to <25 (overweight), from 25 to <30 (obese I), and ≥30 (obese II). Systolic blood pressure (SBP, mmHg), diastolic blood pressure (DBP, mmHg), fasting blood glucose (mg/dL), and total cholesterol (mg/dL) were measured. Histories of benign paroxysmal vertigo (BPPV), vestibular neuronitis (VN), other types of peripheral vertigo, and dyslipidemia were defined based on 2 or more clinical visits.

### 2.6. Statistical Method

The variables were compared between the study (MD or osteoporosis) and the control I or control II groups using standardized differences.

The hazard ratios (HRs) and 95% confidence intervals (CIs) of MD for osteoporosis (study I) and osteoporosis for MD (study II) were estimated using stratified Cox proportional hazard models. All collected variables were adjusted. 

**Figure 2:**
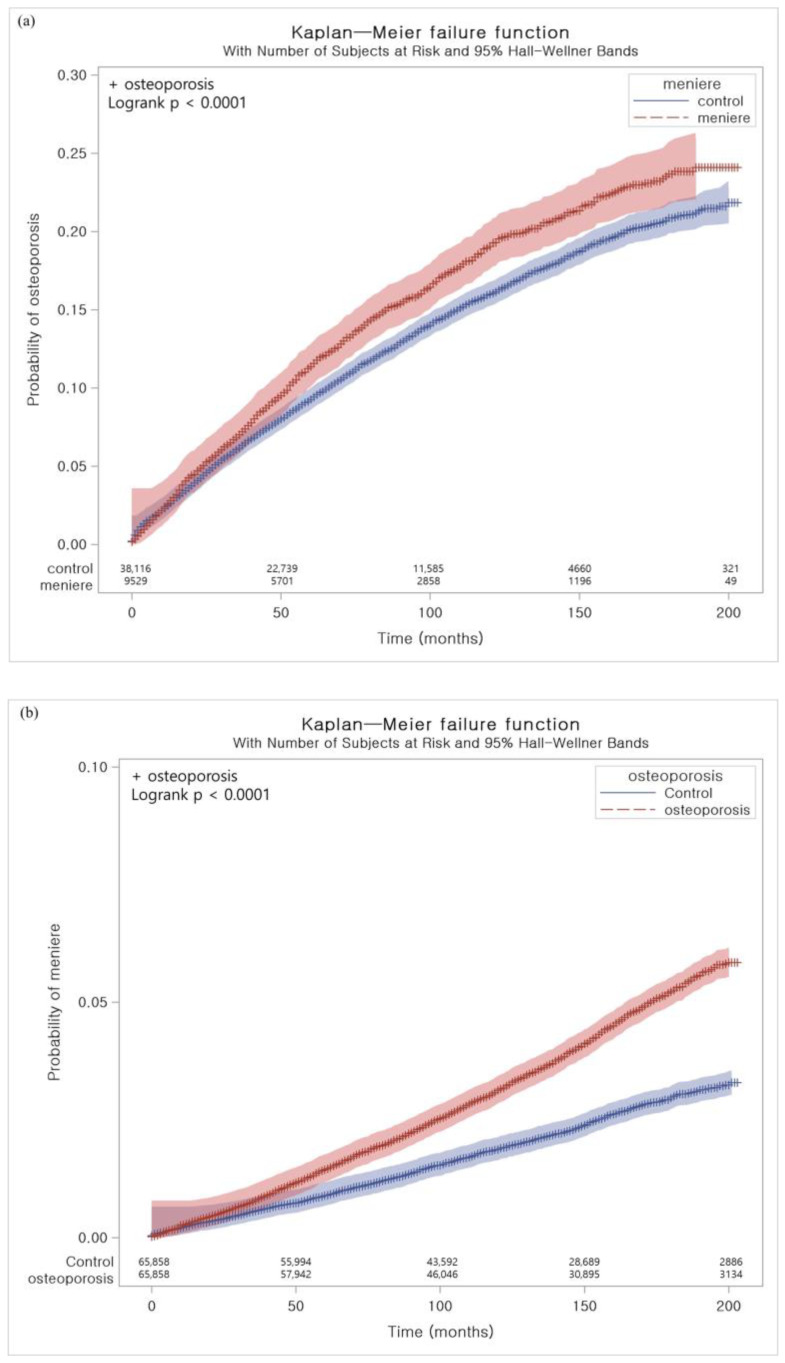
(**a**) Kaplan–Meier curve for the cumulative incidence rates of osteoporosis in Meniere’s disease participants and control I group (**b**) Kaplan–Meier curve for the cumulative incidence rates of Meniere’s disease in osteoporosis participants and control II group.

The cumulative incidence rates of osteoporosis in MD and control I groups (Figure 2a) and those of MD in osteoporosis participants and control II group (Figure 2b) were estimated using the Kaplan–Meier curve and log rank test.

Secondary analyses were conducted according to age, sex, income, region, blood pressure, fasting blood glucose, and total cholesterol. Interaction analyses were conducted to explore the interaction of the variables with MD or osteoporosis. A *p* value < 0.05 was regarded as statistical significance. SAS version 9.4 (SAS Institute Inc., Cary, NC, USA) was utilized.

## 3. Results

In total, 13.3% of MD patients and 11.3% of control I patients had osteoporosis. (sd = 0.06, Table 1). The histories of BPPV, VN, and other types of peripheral vertigo were more frequent in the MD group than in the control I group. The rates of obesity, hyperglycemia, and high CCI were greater in the MD group than in the control I group. On the other hand, current smoking, alcohol consumption, high SBP and DBP, and hypercholesterolemia were greater in the control I group than in the MD group.

**Figure 3:**
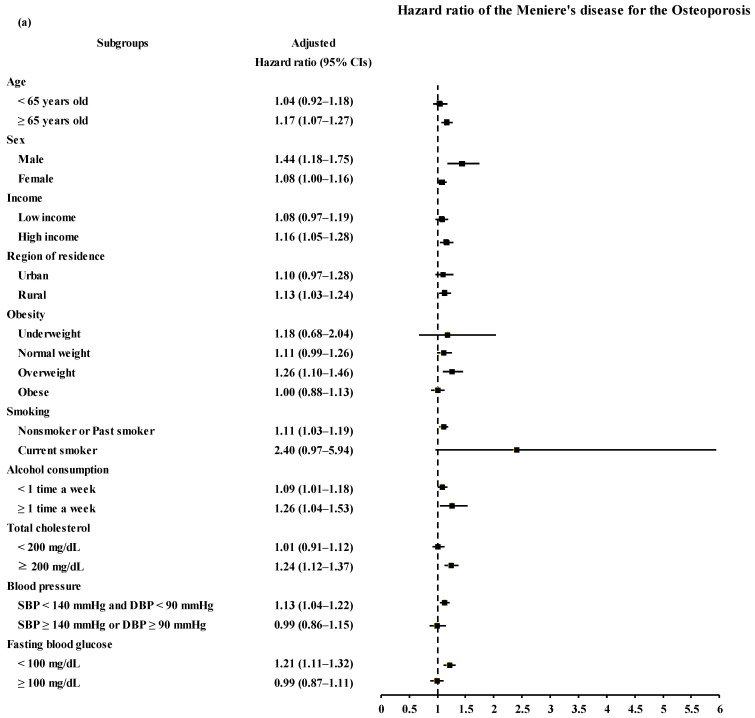

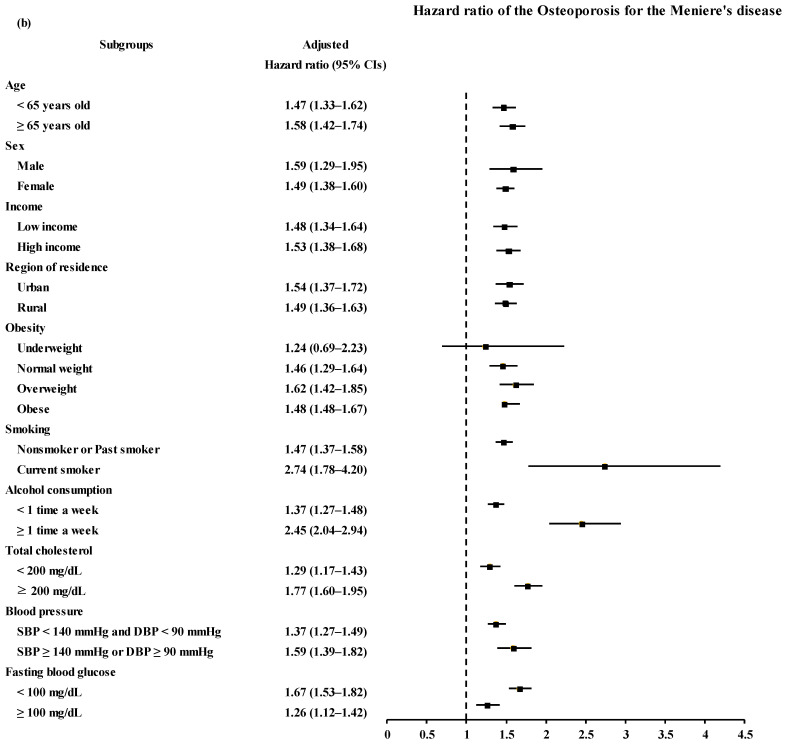
(**a**) Adjusted hazard ratios of osteoporosis in Meniere’s disease patients according to age, sex, income, region, smoking status, alcohol consumption, obesity, blood pressure, fasting blood glucose, and total cholesterol (**b**) Adjusted hazard ratios of Meniere’s disease in osteoporosis patients according to age, sex, income, region, smoking status, alcohol consumption, obesity, blood pressure, fasting blood glucose, and total cholesterol.

The patients with MD demonstrated a higher risk of a previous history of osteoporosis than the control I group (Table 2 and Figure 2a). The adjusted HR for osteoporosis was 1.12 in the MD group (95% CI = 1.04–1.20, *p* = 0.003). Interaction analyses demonstrated significant interactions between MD and sex, smoking, fasting blood glucose, and cholesterol levels. Subgroups of ≥65 years old, men, high income participants, rural residents, nonsmokers or past smokers, overweight participants, and participants with normal blood pressure, normal fasting blood glucose, and high total cholesterol levels presented a consistently higher risk of MD related to osteoporosis (Table 2 and Figure 3a).

In study II, 3.7% of the osteoporosis patients and 2.0% of the control II group had MD (sd = 0.10, Table 3). The histories of BPPV, VN, and other types of peripheral vertigo were more frequent in the osteoporosis group than in the control II group.

MD was associated with an increased risk of osteoporosis (Table 4 and Figure 2b). The patients with osteoporosis had a 1.50 times higher risk of previous MD than the control II group (95% CI = 1.40–1.61, *p* < 0.001). Interaction analyses demonstrated significant interactions between osteoporosis and smoking, alcohol consumption, fast blood glucose, and total cholesterol levels. A higher risk of osteoporosis associated with prior MD was maintained in all subgroups except for the underweight group (Table 4 and Figure 3b).

## 4. Discussion

A previous history of osteoporosis was related to a higher risk of subsequent MD in the present study. On the other hand, a prior history of MD was associated with a greater risk of osteoporosis. In particular, the risk of osteoporosis in patients with MD was as high as 1.50 in multivariable analysis. Thus, a potential risk of osteoporosis should be considered in patients with MD in the clinic. The current results enlarged previous knowledge on the association of MD with osteoporosis by elucidating the temporal relation between the two diseases.

Decreased bone mineral density in patients with MD has been documented [16]. As many as 74% of patients with MD had T-scores less than −1.0, a value found in 39% of the control participants [16]. However, this study was limited due to the small study population (23 MD patients and 23 controls) [16]. Although no other study has evaluated the association between osteoporosis and MD, prior researchers have reported impaired vestibular dysfunction and cochlear impairment in patients with osteoporosis [10,17,18]. The patients with low bone mineral density in the older population demonstrated a 3.72 times (95% CI = 1.07–12.85) higher rate of balance impairment and a 5.30 times (1.20–23.26) higher rate of hearing impairment [17]. The plausible pathophysiologic mechanism involves the fact that bone remodeling can induce resorption of the bony labyrinth and otoconial dislodgement, which will result in dysfunction of the cochleovestibular organ [18].

Patients with MD demonstrated an increased risk of osteoporosis in study II. The altered vestibular function in patients with MD could impact bone remodeling regulation. The vestibular system regulates the equilibrium function via innervation to the brainstem and cerebellum and can influence physical activity and the risk of falls. In addition, the vestibular connection with the brainstem autonomic system was suggested to regulate the cardiovascular function and bone homeostasis via sympathetic nerve regulation [11]. To support this hypothesis, in animal studies, vestibular dysfunction decreased the bone mass, which was prevented with sympathetic blockers or genetic deletion of the adrenergic receptor in osteoblasts [19,20]. Moreover, the increase in bone mineral content according to gravity change was shown to be mediated by vestibular function in a mouse study [21]. In that study, vestibular dysfunction inhibited the growth of bone mass related to hypergravity [21]. Therefore, it can be presumed that the vestibular function may have a crucial role in maintaining the bone mass and that vestibular dysfunction in patients with MD may increase the risk of bone loss and osteoporosis.

Furthermore, patients with osteoporosis reported a high risk of MD (study I). However, the risk was not great in this study (adjusted HR = 1.12). A decreased bone mineral density could increase the risk of otoconial dysfunction, which was suggested as one of the pathophysiologic mechanisms of MD [7]. Detached saccular otoconia obstructing the endolymphatic flow of the inner ear were suggested to induce MD [7,22]. Prior researchers documented a high risk of otoconial detachment or degeneration associated with osteoporosis [23]. Thus, otoconial dislodgement can be one of the possible causes of MD in patients with osteoporosis. Furthermore, the high concentration of free calcium ions in patients with osteoporosis can decrease the capacity of dissolving the dislodged otoconia [24].

The present study analyzed a large nationwide adult population in Korea. Control participants were selected based on matching variables, and selection bias was attenuated by random selection among a large cohort population. The laboratory measured data of SBP, DBP, serum glucose level, and cholesterol level were adjusted, and comorbidities were adjusted using the CCI score. In addition, lifestyle factors of smoking, alcohol consumption, and obesity were examined and adjusted. These variables can be related to osteoporosis and MD. For instance, obesity was suggested to be associated with osteoporosis in a previous study [25]. The variables analyzed in the current study can be further evaluated using a machine learning analysis in order to understand which are the main predictors of osteoporosis in MD. Because the health claim data did not include the results of vestibular and audiometric tests, the type and severity of MD could not be assessed in this study. To attenuate the misdiagnosis of MD and the confounding effect of other vestibular disorders, BPPV, VN, and other types of peripheral vertigo were evaluated. For osteoporosis, dual energy X-ray absorptiometry results and medication histories could not be evaluated. Because asymptomatic patients with osteoporosis can remain undiagnosed before the occurrence of an osteoporotic fracture, a selection bias is possible in our health claim cohort. Patients with MD can be prone to falls, which may mediate the current relationship between osteoporosis and MD. Because there are age- or sex-specific features for both osteoporosis and MD, there may be an age- or sex-specific relationship between osteoporosis and MD. However, the large number of participants in the current study resulted in a significant association between osteoporosis and MD in most subgroups. Forthcoming studies on the impact of the treatment of osteoporosis on MD and on the influence of MD management or types of MD on osteoporosis can solve the current limitations. 

## 5. Conclusions

Patients with MD showed a high rate of subsequent occurrence of osteoporosis. In addition, patients with osteoporosis showed a greater rate of MD occurrence. Clinicians need to consider this reciprocal association when managing patients with MD and osteoporosis.

## Figures and Tables

**Table 1:** General Characteristics of the Participants.

Characteristics		Total Participants		
		Meniere’s Disease (n, %)	Control (n, %)	Standardized Difference
Age (years old)				0.00
	40–44	132 (1.4)	528 (1.4)	
	45–49	547 (5.7)	2188 (5.7)	
	50–54	1230 (12.9)	4920 (12.9)	
	55–59	1986 (20.8)	7944 (20.8)	
	60–64	1738 (18.2)	6952 (18.2)	
	65–69	1519 (15.9)	6076 (15.9)	
	70–74	1189 (12.5)	4756 (12.5)	
	75–79	742 (7.8)	2968 (7.8)	
	80–84	332 (3.5)	1328 (3.5)	
	85+	114 (1.2)	456 (1.2)	
Sex				0.00
	Male	4690 (49.2)	18,760 (49.2)	
	Female	4839 (50.8)	19,356 (50.8)	
Income				0.00
	1 (lowest)	1523 (16.0)	6092 (16.0)	
	2	1202 (12.6)	4808 (12.6)	
	3	1500 (15.7)	6000 (15.7)	
	4	2088 (21.9)	8352 (21.9)	
	5 (highest)	3216 (33.8)	12,864 (33.8)	
Region of residence				0.00
	Urban	4087 (42.9)	16,348 (42.9)	
	Rural	5442 (57.1)	21,768 (57.1)	
Obesity ^†^				0.08
	Underweight	185 (1.9)	889 (2.3)	
	Normal	3017 (31.7)	13,246 (34.8)	
	Overweight	2578 (27.1)	10,333 (27.1)	
	Obese I	3388 (35.6)	12,349 (32.4)	
	Obese II	361 (3.8)	1299 (3.4)	
Smoking status				0.16
	Nonsmoker	7747 (81.3)	31,249 (82.0)	
	Past smoker	1714 (18.0)	5891 (15.5)	
	Current smoker	68 (0.7)	976 (2.6)	
Alcohol consumption				0.06
	<1 time a week	6234 (65.4)	23,822 (62.5)	
	≥1 time a week	3295 (34.6)	14,294 (37.5)	
Systolic blood pressure				0.02
	<120 mmHg	2825 (29.7)	11,661 (30.6)	
	120–139 mmHg	4937 (51.8)	18,669 (49.0)	
	≥140 mmHg	1767 (18.5)	7786 (20.4)	
Diastolic blood pressure				0.12
	<80 mmHg	5438 (57.1)	18,825 (49.4)	
	80–89 mmHg	3123 (32.8)	13,359 (35.1)	
	≥90 mmHg	968 (10.2)	5932 (15.6)	
Fasting blood glucose				0.09
	<100 mg/dL	4870 (51.1)	22,776 (59.8)	
	100–125 mg/dL	3510 (36.8)	11,416 (30.0)	
	≥126 mg/dL	1149 (12.1)	3924 (10.3)	
Total cholesterol				0.16
	<200 mg/dL	5671 (59.5)	20,579 (54.0)	
	200–239 mg/dL	2724 (28.6)	12,362 (32.4)	
	≥240 mg/dL	1134 (11.9)	5175 (13.6)	
CCI score				0.14
	0	5499 (57.7)	24,021 (63.0)	
	1	1860 (19.5)	5947 (15.6)	
	≥2	2170 (22.8)	8148 (21.4)	
Dyslipidemia		6081 (63.8)	20,422 (53.6)	0.21
Benign paroxysmal vertigo		4607 (48.4)	4772 (12.5)	0.85
Vestibular neuronitis		1770 (18.6)	1169 (3.1)	0.52
Other peripheral vertigo		3582 (37.6)	3587 (9.4)	0.70
Osteoporosis		1266 (13.3)	4287 (11.3)	0.06

Abbreviations: CCI, Charlson comorbidity index. SD, standard deviation. ^†^ Obesity (BMI, body mass index, kg/m^2^) was categorized as <18.5 (underweight), from 18.5 to <23 (normal), from 23 to <25 (overweight), from 25 to <30 (obese I), and ≥30 (obese II).

**Table 2:** Crude and adjusted hazard ratios of Meniere’s disease for osteoporosis in subgroups according to age, sex, income, region, smoking status, alcohol consumption, obesity, blood pressure, fasting blood glucose, and total cholesterol.

	N of Event/ N of Total (%)	F/U Duration (PY)	IR per 1000 (PY)	IRD (95% CI)	Hazard Ratios				
					Crude	*p*-Value	Adjusted ^†^	*p*-Value	*p* for Interaction
**Total**									
Meniere’s disease	1266/9529 (13.3)	55,280	22.90	3.59 (2.27 to 4.91)	1.20 (1.13–1.28)	<0.001 *	1.12 (1.04–1.20)	0.003 *	
Control	4287/38,116 (11.2)	221,976	19.31	3.59 (2.27 to 4.91)	1		1		
Age < 65 years old									0.211
Meniere’s disease	440/3895 (11.3)	29,322	15.01	1.45 (−0.05 to 2.96)	1.12 (1.01–1.24)	0.037 *	1.04 (0.92–1.18)	0.533	
Control	1595/15,580 (10.2)	117,693	13.55		1		1		
Age ≥ 65 years old									
Meniere’s disease	826/5634 (14.7)	25,958	31.82	6.01 (3.77 to 8.24)	1.25 (1.15–1.35)	<0.001 *	1.17 (1.07–1.27)	<0.001 *	
Control	2692/22,536 (11.9)	104,283	25.81		1		1		
Male									0.001 *
Meniere’s disease	194/4690 (4.1)	26,878	7.22	2.61 (1.66 to 3.57)	1.56 (1.32–1.85)	<0.001 *	1.44 (1.18–1.75)	<0.001 *	
Control	487/18,760 (2.6)	105,790	4.60		1		1		
Female									
Meniere’s disease	1072/4839 (22.2)	28,402	37.74	5.04 (2.66 to 7.42)	1.15 (1.08–1.23)	<0.001 *	1.08 (1.00–1.16)	0.060	
Control	3800/19,356 (19.6)	116,186	32.71		1		1		
Low income									0.516
Meniere’s disease	629/4225 (14.9)	24,633	25.53	3.74 (1.64 to 5.84)	1.18 (1.08–1.30)	<0.001 *	1.08 (0.97–1.19)	0.166	
Control	2145/16,900 (12.7)	98,415	21.80		1		1		
High income									
Meniere’s disease	637/5304 (12.0)	30,647	20.79	3.45 (1.77 to 5.13)	1.22 (1.11–1.33)	<0.001 *	1.16 (1.05–1.28)	0.005 *	
Control	2142/21,216 (10.1)	123,561	17.34		1		1		
Urban residents									0.410
Meniere’s disease	467/4087 (11.4)	24,815	18.82	2.19 (0.37 to 4.01)	1.14 (1.03–1.26)	0.013 *	1.10 (0.97–1.24)	0.126	
Control	1638/16,348 (10.0)	98,485	16.63		1		1		
Rural residents									
Meniere’s disease	799/5442 (14.7)	30,465	26.23	4.78 (2.90 to 6.65)	1.24 (1.14–1.34)	<0.001 *	1.13 (1.03–1.24)	<0.001 *	
Control	2649/21,768 (12.2)	123,491	21.45		1		1		
Nonsmoker or Past smoker									0.002 *
Meniere’s disease	1247/9461 (13.2)	54,747	22.78	2.96 (1.62 to 4.30)	1.19 (1.12–1.27)	<0.001 *	1.11 (1.03–1.19)	0.006 *	
Control	4209/37,140 (11.3)	212,367	19.82		1		1		
Current smoker									
Meniere’s disease	19/68 (27.9)	533	35.65	27.53 (19.00 to 36.06)	2.71 (1.33–5.56)	0.006 *	2.40 (0.97–5.94)	0.057	
Control	78/976 (8.0)	9609	8.12		1		1		
Alcohol consumption < 1 time a week									0.375
Meniere’s disease	1078/6234 (17.3)	39,023	27.62	2.92 (1.15 to 4.69)	1.19 (1.11–1.27)	<0.001 *	1.09 (1.01–1.18)	0.026	
Control	3714/23,822 (15.6)	150,315	24.71		1		1		
Alcohol consumption ≥ 1 time a week									
Meniere’s disease	188/3295 (5.7)	16,257	11.56	3.57 (1.98 to 5.15)	1.28 (1.08–1.51)	0.005 *	1.26 (1.04–1.53)	0.018	
Control	573/14,294 (4.0)	71,661	8.00		1		1		
Underweight									0.123
Meniere’s disease	40/185 (21.6)	939	42.60	15.79 (3.66 to 27.92)	1.52 (0.99–2.34)	0.055	1.18 (0.68–2.04)	0.555	
Control	117/889 (13.2)	4364	26.81		1		1		
Normal weight									
Meniere’s disease	439/3017 (14.6)	17,402	25.23	4.63 (2.22 to 7.03)	1.21 (1.09–1.35)	<0.001 *	1.11 (0.99–1.26)	0.083	
Control	1593/13,246 (12.0)	77,328	20.60		1		1		
Overweight									
Meniere’s disease	352/2578 (13.7)	14,667	24.00	5.96 (3.47 to 8.46)	1.35 (1.19–1.52)	<0.001 *	1.26 (1.10–1.46)	0.001 *	
Control	1102/10,333 (10.7)	61,097	18.04		1		1		
Obese									
Meniere’s disease	435/3749 (11.6)	22,272	19.53	0.90 (−1.14 to 2.94)	1.10 (0.99–1.23)	0.082	1.00 (0.88–1.13)	0.955	
Control	1475/13,648 (10.8)	79,187	18.63		1		1		
SBP < 140 mmHg and DBP < 90 mmHg									0.604
Meniere’s disease	973/7530 (12.9)	43,501	22.37	3.13 (1.64 to 4.62)	1.21 (1.13–1.30)	<0.001 *	1.13 (1.04–1.22)	0.005 *	
Control	3141/28,886 (10.9)	163,298	19.23		1		1		
SBP ≥ 140 mmHg or DBP ≥ 90 mmHg									
Meniere’s disease	293/1999 (14.7)	11,779	24.87	5.34 (2.52 to 8.17)	1.15 (1.01–1.31)	0.037	0.99 (0.86–1.15)	0.922	
Control	1146/9230 (12.4)	58,678	19.53		1		1		
Fasting blood glucose < 100 mg/dL									<0.001 *
Meniere’s disease	785/4870 (16.1)	27,581	28.46	7.93 (6.03 to 9.84)	1.37 (1.26–1.48)	<0.001 *	1.21 (1.11–1.32)	<0.001 *	
Control	2910/22,776 (12.8)	141,761	20.53		1		1		
Fasting blood glucose ≥ 100 mg/dL									
Meniere’s disease	481/4659 (10.3)	27,699	17.37	0.20 (−1.59 to 1.99)	1.04 (0.94–1.15)	0.478	0.99 (0.87–1.11)	0.827	
Control	1377/15,340 (9.0)	80,215	17.17		1		1		
Total cholesterol < 200 mg/dL									<0.001 *
Meniere’s disease	629/5671 (11.1)	33,282	18.90	1.40 (−0.22 to 3.03)	1.07 (0.98–1.17)	0.141	1.01 (0.91–1.12)	0.800	
Control	2068/20,579 (10.0)	118,204	17.50		1		1		
Total cholesterol ≥ 200 mg/dL									
Meniere’s disease	637/3858 (16.5)	21,998	28.96	7.57 (5.38 to 9.77)	1.36 (1.25–1.49)		1.24 (1.12–1.37)	<0.001 *	
Control	2219/17,537 (12.7)	103,772	21.38		1		1		

Abbreviation: IR, incidence rate; IRD, incidence rate difference; SBP, systolic blood pressure; DBP, diastolic blood pressure; PY. person-year; * Significance at *p* < 0.05. ^†^ Adjusted for age, sex, income, region of residence, SBP, DBP, fasting blood glucose, total cholesterol, obesity, smoking, alcohol consumption, CCI scores, dyslipidemia, benign paroxysmal vertigo, vestibular neuronitis, and other types of peripheral vertigo.

**Table 3:** General Characteristics of the Participants.

Characteristics		Total Participants		
		Osteoporosis (n, %)	Control (n, %)	Standardized Difference
Age (years old)				0.00
	40–44	1000 (1.5)	1000 (1.5)	
	45–49	5010 (7.6)	5010 (7.6)	
	50–54	11,850 (18.0)	11,850 (18.0)	
	55–59	14,944 (22.7)	14,944 (22.7)	
	60–64	13,199 (20.0)	13,199 (20.0)	
	65–69	6950 (10.6)	6950 (10.6)	
	70–74	6607 (10.0)	6607 (10.0)	
	75–79	4229 (6.4)	4229 (6.4)	
	80–84	1731 (2.6)	1731 (2.6)	
	85+	338 (0.5)	338 (0.5)	
Sex				0.00
	Male	11,749 (17.8)	11,749 (17.8)	
	Female	54,109 (82.2)	54,109 (82.2)	
Income				
	1 (lowest)	12,421 (18.9)	12,421 (18.9)	
	2	9987 (15.2)	9987 (15.2)	
	3	10,829 (16.4)	10,829 (16.4)	
	4	13,376 (20.3)	13,376 (20.3)	
	5 (highest)	19,245 (29.2)	19,245 (29.2)	
Region of residence				0.00
	Urban	27,896 (42.4)	27,896 (42.4)	
	Rural	37,962 (57.6)	37,962 (57.6)	
Obesity ^†^				0.16
	Underweight	2826 (4.3)	1545 (2.4)	
	Normal	25,405 (38.6)	22,504 (34.2)	
	Overweight	16,515 (25.1)	17,308 (26.3)	
	Obese I	18,836 (28.6)	21,581 (32.8)	
	Obese II	2276 (3.5)	2920 (4.4)	
Smoking status				0.20
	Nonsmoker	58,740 (89.2)	58,076(88.2)	
	Past smoker	5150 (7.8)	3388(5.1)	
	Current smoker	1968 (3.0)	4394(6.7)	
Alcohol consumption				
	<1 time a week	55,978 (85.0)	53,924(81.9)	
	≥1 time a week	9880 (15.0)	11,934(18.1)	
Systolic blood pressure				0.04
	<120 mmHg	20,144 (30.6)	19,771(30.0)	
	120–139 mmHg	32,475 (49.3)	29,094(44.2)	
	≥140 mmHg	20,144 (30.6)	16,993(25.8)	
Diastolic blood pressure				0.25
	<80 mmHg	39,450 (59.9)	30,318(46.0)	
	80–89 mmHg	19,893 (30.2)	22,020(33.4)	
	≥90 mmHg	6515 (9.9)	13,520(20.5)	
Fasting blood glucose				0.04
	<100 mg/dL	37,846 (57.5)	42,460 (64.5)	
	100–125 mg/dL	21,848 (33.2)	17,168 (26.1)	
	≥126 mg/dL	6164 (9.4)	6230 (9.5)	
Total cholesterol				0.23
	<200 mg/dL	37,697 (57.2)	31,720 (48.2)	
	200–239 mg/dL	19,550 (29.7)	22,908 (34.8)	
	≥240 mg/dL	8611 (13.1)	11,230 (17.1)	
CCI score				0.13
	0	36,787 (55.9)	40,072 (60.9)	
	1	12,023 (18.3)	10,153 (15.4)	
	≥2	17,048 (25.9)	15,633 (23.7)	
Dyslipidemia		41,037 (62.3)	33,512 (50.9)	0.23
Benign paroxysmal vertigo		13,651 (20.7)	9551 (14.5)	0.16
Vestibular neuronitis		3646 (5.5)	2426 (3.7)	0.09
Other peripheral vertigo		10,765 (16.4)	7325 (11.1)	0.15
Meniere’s disease		2441 (3.7)	1339 (2.0)	0.10

Abbreviations: CCI, Charlson comorbidity index. SD, standard deviation. ^†^ Obesity (BMI, body mass index, kg/m^2^) was categorized as <18.5 (underweight), from 18.5 to <23 (normal), from 23 to <25 (overweight), from 25 to <30 (obese I), and ≥30 (obese II).

**Table 4:** Crude and adjusted hazard ratios of osteoporosis for osteoporosis in subgroups according to age, sex, income, region, smoking status, alcohol consumption, obesity, blood pressure, fasting blood glucose, and total cholesterol.

	N of Event/ N of Total (%)	F/U Duration (PY)	IR per 1000 (PY)	IRD (95% CI)	Hazard Ratios				
					Crude	*p*-Value	Adjusted ^†^	*p*-Value	*p* for Interaction
Total									
Osteoporosis	2441/65,858 (3.7)	686,328	3.56	1.51 (1.34 to 1.69)	1.74 (1.63–1.86)	<0.001 *	1.50 (1.40–1.61)	<0.001 *	
Control	1339/65,858 (2.0)	655,747	2.04		1		1		
Age < 65 years old									
Osteoporosis	1171/32,804 (3.6)	365,188	3.21	1.26 (1.02 to 1.49)	1.64 (1.50–1.81)	<0.001 *	1.47 (1.33–1.62)	<0.001 *	0.310
Control	706/32,804 (2.2)	362,009	1.95		1		1		
Age ≥ 65 years old									
Osteoporosis	1270/33,054 (3.8)	321,140	3.95	1.80 (1.52 to 2.08)	1.85 (1.68–2.03)	<0.001 *	1.58 (1.42–1.74)	<0.001 *	
Control	633/33,054 (1.9)	293,738	2.15		1		1		
Male									0.223
Osteoporosis	308/11,749 (2.6)	83,587	3.68	1.68 (1.16 to 2.19)	1.84 (1.52–2.22)	<0.001 *	1.59 (1.29–1.95)	<0.001 *	
Control	162/11,749 (1.4)	80,678	2.01		1		1		
Female									
Osteoporosis	2133/54,109 (3.9)	602,741	3.54	1.49 (1.30 to 1.68)	1.73 (1.61–1.86)	<0.001 *	1.49 (1.38–1.60)	<0.001 *	
Control	1177/54,109 (2.2)	575,069	2.05		1		1		
Low income									0.730
Osteoporosis	1223/33,237 (3.7)	344,847	3.55	1.53 (1.28 to 1.79)	1.76 (1.60–1.94)	<0.001 *	1.48 (1.34–1.64)	<0.001 *	
Control	659/33,237 (2.0)	327,587	2.01		1		1		
High income									
Osteoporosis	1218/32,621 (3.7)	341,481	3.57	1.49 (1.24 to 1.75)	1.72 (1.57–1.89)	<0.001 *	1.53 (1.38–1.68)	<0.001 *	
Control	680/32,621 (2.1)	328,160	2.07		1		1		
Urban residents									0.543
Osteoporosis	986/27,896 (3.5)	294,122	3.35	1.45 (1.18 to 1.71)	1.76 (1.58–1.95)	<0.001 *	1.54 (1.37–1.72)	<0.001 *	
Control	543/27,896 (1.9)	284,889	1.91		1		1		
Rural residents									
Osteoporosis	1455/37,962 (3.8)	392,206	3.71	1.56 (1.32 to 1.81)	1.73 (1.59–1.89)	<0.001 *	1.49 (1.36–1.63)	<0.001 *	
Control	796/37,962 (2.1)	370,858	2.15		1		1		
Nonsmoker or Past smoker									<0.001 *
Osteoporosis	2381/63,890 (3.7)	671,791	3.54	1.46 (1.28 to 1.65)	1.70 (1.59–1.82)	<0.001 *	1.47 (1.37–1.58)	<0.001 *	
Control	1289/61,464 (2.1)	619,678	2.08		1		1		
Current smoker									
Osteoporosis	60/1968 (3.0)	14,537	4.13	2.74 (1.84 to 3.64)	3.03 (2.05–4.47)	<0.001 *	2.74 (1.78–4.20)	<0.001 *	
Control	50/4394 (1.1)	36,069	1.39		1		1		
Alcohol consumption < 1 time a week									<0.001 *
Osteoporosis	1898/55,978 (3.4)	593,096	3.20	1.16 (0.98 to 1.35)	1.57 (1.46–1.69)	<0.001 *	1.37 (1.27–1.48)	<0.001 *	
Control	1161/53,924 (2.2)	569,883	2.04		1		1		
Alcohol consumption ≥ 1 time a week									
Osteoporosis	543/9880 (5.5)	93,232	5.82	3.75 (3.16 to 4.34)	2.83 (2.38–3.37)	<0.001 *	2.45 (2.04–2.94)	<0.001 *	
Control	178/11,934 (1.5)	85,864	2.07		1		1		
Under weight									0.712
Osteoporosis	63/2826 (2.2)	27,050	2.33	0.92 (−0.03 to 1.87)	1.56 (0.92–2.66)	0.101	1.24 (0.69–2.23)	0.471	
Control	18/1545 (1.2)	12,782	1.41		1		1		
Normal weight									
Osteoporosis	893/25,405 (3.5)	257,394	3.47	1.50 (1.20 to 1.80)	1.74 (1.55–1.95)	<0.001 *	1.46 (1.29–1.64)	<0.001 *	
Control	426/22,504 (1.9)	216,567	1.97		1		1		
Overweight									
Osteoporosis	672/16,515 (4.1)	172,858	3.89	1.71 (1.35 to 2.08)	1.78 (1.57–2.02)	<0.001 *	1.62 (1.42–1.85)	<0.001 *	
Control	376/17,308 (2.2)	172,944	2.17		1		1		
Obese									
Osteoporosis	813/21,112 (3.9)	229,026	3.55	1.50 (1.21 to 1.80)	1.73 (1.55–1.93)	<0.001 *	1.48 (1.32–1.67)	<0.001 *	
Control	519/24,501 (2.1)	253,454	2.05		1		1		
SBP < 140 mmHg and DBP < 90 mmHg									0.076
Osteoporosis	1884/51,259 (3.7)	529,039	3.56	1.44 (1.23 to 1.66)	1.68 (1.55–1.81)	<0.001 *	1.37 (1.27–1.49)	<0.001 *	
Control	947/46,032 (2.1)	447,432	2.12		1		1		
SBP ≥ 140 mmHg or DBP ≥ 90 mmHg									
Osteoporosis	557/14,599 (3.8)	157,289	3.54	1.66 (1.33 to 1.99)	1.87 (1.64–2.13)	<0.001 *	1.59 (1.39–1.82)	<0.001 *	
Control	392/19,826 (2.0)	208,315	1.88		1		1		
Fasting blood glucose < 100 mg/dL									<0.001 *
Osteoporosis	1517/37,846 (4.0)	389,966	3.89	1.88 (1.65 to 2.11)	1.95 (1.79–2.11)	<0.001 *	1.67 (1.53–1.82)	<0.001 *	
Control	879/42,460 (2.1)	437,869	2.01		1		1		
Fasting blood glucose ≥ 100 mg/dL									
Osteoporosis	924/28,012 (3.3)	296,362	3.12	1.01 (0.72 to 1.29)	1.48 (1.32–1.66)	<0.001 *	1.26 (1.12–1.42)	<0.001 *	
Control	460/23,398 (2.0)	217,878	2.11		1		1		
Total cholesterol < 200 mg/dL									<0.001 *
Osteoporosis	1287/37,697 (3.4)	393,783	3.27	1.18 (0.93 to 1.42)	1.52 (1.38–1.68)	<0.001 *	1.29 (1.17–1.43)	<0.001 *	
Control	641/31,720 (2.0)	306,281	2.09		1		1		
Total cholesterol ≥ 200 mg/dL									
Osteoporosis	1154/28,161 (4.1)	292,545	3.94	1.95 (1.68 to 2.21)	2.04 (1.86–2.24)	<0.001 *	1.77 (1.60–1.95)	<0.001 *	
Control	698/34,138 (2.0)	349,466	2.00		1		1		

Abbreviation: IR, incidence rate; IRD, incidence rate difference; SBP, systolic blood pressure; DBP, diastolic blood pressure; PY. person-year; * Significance at *p* < 0.05. ^†^ Adjusted for age, sex, income, region of residence, SBP, DBP, fasting blood glucose, total cholesterol, obesity, smoking, alcohol consumption, CCI scores, dyslipidemia, benign paroxysmal vertigo, vestibular neuronitis, and other types of peripheral vertigo.

## Data Availability

Releasing of the data by the researcher is not legally permitted. All data are available from the database of the Korea Centers for Disease Control and Prevention. The Korea Centers for Disease Control and Prevention allows data access, at a particular cost, for any researcher who promises to follow the research ethics. The data of this article can be downloaded from the website after agreeing to follow the research ethics.
